# BolA-like protein (IbaG) promotes biofilm formation and pathogenicity of *Vibrio parahaemolyticus*

**DOI:** 10.3389/fmicb.2024.1436770

**Published:** 2024-07-31

**Authors:** Wenchao Wang, Yangyang Li, Shuqi Lu, Pengxuan Liu, Xiangan Han, Weidong Sun, Quan Wang, Weihuan Fang, Wei Jiang

**Affiliations:** ^1^Shanghai Veterinary Research Institute, Chinese Academy of Agricultural Sciences, Shanghai, China; ^2^College of Veterinary Medicine, Nanjing Agricultural University, Nanjing, China; ^3^Zhejiang Provincial Key Laboratory of Preventive Veterinary Medicine, Institute of Preventive Veterinary Medicine, Zhejiang University, Hangzhou, Zhejiang, China

**Keywords:** motility, biofilm formation, bacterial pathogenicity, *Vibrio parahaemolyticus*, IbaG

## Abstract

*Vibrio parahaemolyticus* is a gram-negative halophilic bacterium widespread in temperate and tropical coastal waters; it is considered to be the most frequent cause of *Vibrio*-associated gastroenteritis in many countries. BolA-like proteins, which reportedly affect various growth and metabolic processes including flagellar synthesis in bacteria, are widely conserved from prokaryotes to eukaryotes. However, the effects exerted by BolA-like proteins on *V. parahaemolyticus* remain unclear, and thus require further investigation. In this study, our purpose was to investigate the role played by BolA-like protein (IbaG) in the pathogenicity of *V. parahaemolyticus*. We used homologous recombination to obtain the deletion strain Δ*ibaG* and investigated the biological role of BolA family protein IbaG in *V. parahaemolyticus*. Our results showed that IbaG is a bacterial transcription factor that negatively modulates swimming capacity. Furthermore, overexpressing IbaG enhanced the capabilities of *V. parahaemolyticus* for swarming and biofilm formation. In addition, inactivation of *ibaG* in *V. parahaemolyticus* SH112 impaired its capacity for colonizing the heart, liver, spleen, and kidneys, and reduced visceral tissue damage, thereby leading to diminished virulence, compared with the wild-type strain. Finally, RNA-sequencing revealed 53 upregulated and 71 downregulated genes in the deletion strain Δ*ibaG*. KEGG enrichment analysis showed that the two-component system, quorum sensing, bacterial secretion system, and numerous amino acid metabolism pathways had been altered due to the inactivation of *ibaG*. The results of this study indicated that IbaG exerts a considerable effect on gene regulation, motility, biofilm formation, and pathogenicity of *V. parahaemolyticus*. To the best of our knowledge, this is the first systematic study on the role played by IbaG in *V. parahaemolyticus* infections. Thus, our findings may lead to a better understanding of the metabolic processes involved in bacterial infections and provide a basis for the prevention and control of such infections.

## 1 Introduction

*Vibrio parahaemolyticus*, first identified in Japan in 1950, is the main cause of vibriosis outbreaks that negatively affect global aquaculture, resulting in food borne diseases and huge economic losses (Velazquez-Roman et al., 2014). *V. parahaemolyticus* infections may result in diverse clinical symptoms, ranging from common wound infections to acute gastroenteritis to life-threatening septicemia (Broberg et al., 2011). The pathogenicity of *V. parahaemolyticus* is closely associated with numerous virulence factors, such as motility, biofilm formation, hemolysin, the type VI secretion system (T6SS), and lipopolysaccharides (Li et al., 2019). Bacterial virulence factors, which are the principal molecules involved in bacterial infection, and colonization facilitates the spread of microorganisms within the host (Cross, 2008; Li et al., 2019). Therefore, gaining an in-depth understanding of the mechanisms underlying the pathogenicity of *V. parahaemolyticus* is critical to control its spread.

Infection of a host by pathogens is closely linked to their virulence effectors. The transcriptional regulatory network plays a central role in modulating the tandem expression of such virulence effectors (Shen and Fang, 2012). BolA-like proteins are a widely conserved family present in both prokaryotes and eukaryotes (Moreira et al., 2017). The BolA protein family contains two members, BolA and IbaG, which have been found in some pathogenic bacteria, such as *Vibrio cholerae*, *V. parahaemolyticus* and *Escherichia coli* (da Silva et al., 2023). BolA-like proteins possess a class II KH fold associated with OsmC over oxidoreductase and contain a helix-turn-helix domain that reportedly binds to DNA and regulates transcription (Xue et al., 2022). IbaG has a similar genomic background in *E. coli*, *V. cholerae*, and *V. parahaemolyticus*. MlaBCDEF, which is upstream of IbaG, encodes an ABC transport system that maintains outer membrane lipid asymmetry, and murA, which catalyzes evolutionary group transfers as a part of the first step in peptidoglycan biosynthesis (Fleurie et al., 2019). BolA binds to certain promoters to exert transcriptional regulation, but IbaG does not interact with the same DNA regions (Guinote, 2012). Thus, although BolA and IbaG belong to the same family, their functions differ.

Several studies have revealed the critical role of BolA as a transcriptional regulator of bacteriological responses to environmental stress and survival (Dressaire et al., 2015; Moreira et al., 2017). In *Salmonella enterica* var. Typhimurium, a lack of *bolA* leads to defective virulence, whereas the presence of BolA protects against acidic and oxidative stress and ensures bacterial survival at later stages of growth, particularly under harsh environmental conditions (Mil-Homens et al., 2018). *Klebsiella pneumoniae* BolA has also been recognized as a virulence factor. Deletion of *bolA* facilitates the reduction of microbial load in mouse guts while negatively regulating toxicity-related metabolites, such as biotin and guanosine, thereby playing an important role in pathogenesis (Zhang et al., 2022). The *E. coli* BolA protein regulates the expression of di-guanosine cyclase and phosphodiesterase, which interact with c-di-GMP to regulate flagellar production, motility, and biofilm formation and to enable bacterial transition between the planktonic and adherent phases (Dressaire et al., 2015). IbaG, which is induced under acidic stress, plays a role in Fe-S cluster assembly and transport in *E. coli* (Dlouhy et al., 2016). In *V. cholerae*, IbaG controls *V. cholerae* cell shape and cell envelope homeostasis through its effects on ferrithionein and related pathways (Fleurie et al., 2019). *V. parahaemolyticus*, which belongs to the same genus as *V. cholerae*, is closely linked to human diseases. At present, the function of *bolA* has been well estimated in previous studies, however, little is known about the contribution of another member of the BolA protein family, IbaG, especially the contribution of *ibaG* to the pathogenicity and host adaptation of *V. parahaemolyticus*.

In the current study, we used the homologous recombination technique to obtain the deletion strain Δ*ibaG* to investigate the biological role played by the BolA family protein IbaG in the motility, biofilm formation, and virulence of *V. parahaemolyticus*. Our findings may provide novel insights into the function of IbaG proteins and help generate fresh ideas aimed at the prevention and control of *V. parahaemolyticus.*

## 2 Materials and methods

### 2.1 Bacterial strains, plasmids, and growth conditions

All bacterial strains and primers used for functional analysis are listed in Tables 1, 2. *V. parahaemolyticus* strain SH112 (GenBank: JACYGZ000000000.1) was used as the wild-type (WT) strain. This strain was isolated from a clinical specimen and stored in China General Microbial Culture Collection Center under the accession number of CGMCC 1.90013. The *E. coli* strains were cultured in Luria–Bertani (LB) at 37°C. *V. parahaemolyticus* SH112 (Li et al., 2022) and derivatives (mutants, complementary, and *ibaG* overexpression) were grown in LB broth supplemented with 3% NaCl at 37°C with aeration. Plasmid pYAK1 and pMMB207 were used to construct gene-deletion mutants and complementary strains.

**TABLE 1:** Bacterial strains used in this study.

Name	Strain	Resource
****V*.*parahaemolyticus** strains**		
WT	*V. parahaemolyticus* SH112 with empty pMMB207	This study
WT(*ibaG*)	*V. parahaemolyticus* harboring pMMB207-*ibaG*	This study
Δ*ibaG*	Mutation in ibaG gene of strain SH112 *V. Parahaemolyticus*Δ*ibaG* with empty pMMB207	This study
CΔ*ibaG*	*V. Parahaemolyticus*Δ*ibaG* harboring pMMB207-*ibaG*	This study
****E*.*coli** strains**		
CC118 λpir	Λpir lysogen of CC118 Δ (ara-leu) araD ΔlacX74 galE galK phoA20 thi-1 rpsE rpoB argE (Am) recA1	Wang et al., 2007
**Plasmids**		
pYAK1	A suicide vector with ori R6K sacB; Cmr	Ono et al., 2006
pMMB207	RSF1010 derivative, IncQ lacI q Cmr Ptac oriT	Zhou et al., 2008

**TABLE 2:** List of primers used in the study.

Number	Name	Sequence	Purpose
1	*ibaG*-A	TAGAAAGGATCCCTAAGCGTATTACCAGC	Deletion of *ibaG*
2	*ibaG*-B	AATTTACCCCTGATAATTCTTTATGTG	
3	*ibaG*-C	TATCAGGGGTAAATTGGTTTATTGATGGAAA	
4	*ibaG*-D	ATAAAACCCGGG GAAATCCGCCGTATCG	
5	*ibaG*-E	GTGACATTTGCCCGACTA	Test deletion of *ibaG*
6	*ibaG*-F	GCCTGTCTCCACCAAT	
7	*ibaG-*pMMB-F	CTCAAAGGATCCGTGGACAGCGCAAAAGTACA	Clone *ibaG* in pMMB207
8	*ibaG-*pMMB-R	TTTAAGCTGCAGTTACAGCGACATCAGTTTCT	
9	*ibaG*-RT-F	AAGTGGTTGCTGTTGATGCTTGTTTC	RT-qPCR analysis
10	*ibaG*-RT-R	GTGAATGTCGTTACGCTGGATGTATTC	

Restriction sites are underlined.

### 2.2 Construction of *ibaG*-knockout strain and complemented strains

We constructed *ibaG*-knockout strains (Table 1) via homologous recombination, with specific primers for the upstream and downstream segments of *ibaG* being designed and amplified (primers 1–4; Table 2). The obtained upstream and downstream fragments were fused and amplified, following which the suicide plasmid pYAK1 and the fusion fragment were double digested using *Bam* HI and *Sma* I and subsequently introduced into *E. coli* CC118λpir. This was later transferred to the WT *V. parahaemolyticus* and selected in LB medium containing 20% sucrose and the highly selective thiosulfate-citrate-bile-salts-sucrose-agar (TCBS) culture medium with chloramphenicol (10 μg/mL). Following continuous cultivation, strains that did not grow on TCBS plates but grew on sucrose plates were selected for PCR validation and screening for strains with *ibaG* deletions (primers 5 and 6; Table 2).

Primers 7 and 8 (Table 2) were used to amplify a DNA fragment containing *V. parahaemolyticus ibaG*, following which the amplified PCR products and pMMB207 vector were double digested using the restriction enzymes *Bam* HI and *Pst* I. The two were lighted and transformed into *V. parahaemolyticus* and *V. parahaemolyticus*Δ*ibaG* strains, respectively, yielding strains WT (*ibaG*) and CΔ*ibaG* (Table 1).

### 2.3 Analysis of growth curves

The strains WT, WT (*ibaG*), Δ*ibaG* and CΔ*ibaG* were stored in LB medium containing 3% NaCl + 25% glycerol at −80°C. Bacterial cultures were cultivated overnight in 3% NaCl-LB medium at 37°C with 180 rpm agitation. Bacterial cells were diluted with 1:100 (v:v) in 3% NaCl-LB medium at 37°C. The OD_600_ of *V. parahaemolyticus* strains was measured by spectrophotometer (Thermo Fisher Scientific) at hourly intervals. The values were analyzed, and the growth curves of each strain plotted. The experiments were conducted in triplicate.

### 2.4 Analysis of swimming and swarming motility

Swimming and swarming media were prepared as described previously (Noh et al., 2015; Gu et al., 2019). After each strain of WT, WT (*ibaG*), Δ*ibaG* and CΔ*ibaG* was grown at 37°C to an OD_600_ nm of 0.2, 2 μL of each strain was added to the agar plate in vertical drops. After inoculation, swimming plates containing 2% sodium chloride and 0.3% agar were incubated for 4 h at 37°C. Swarming plates containing 2% sodium chloride and 1.5% agar were incubated for 24 h at 30°C to observe the effects of *ibaG* on the motility of *V. parahaemolyticus*. The diameters of the swimming and swarming movements formed by each strain were measured, and the tests were repeated thrice.

### 2.5 Biofilm formation assay

Biofilm thickness in the microtiter plates was determined using a crystal violet assay, as described previously (Naves et al., 2008). Briefly, WT, WT (*ibaG*), Δ*ibaG* and CΔ*ibaG* were cultured in 3% NaCl-LB medium to a final concentration of 10^8^ colony-forming units (CFU)/mL. Following which 200 μL of each strain was transferred to a 96-well polystyrene microtiter plate (Corning, NY, USA) and incubated for 48 h at 30°C. The bacterial solution was discarded after 48 h of culture and washed with phosphate-buffered saline (PBS) two or three times to remove the floating cells. The plates were dried at 37°C for 15 min, and the biofilm cells were stained with 200 μL 1% crystal violet solution (Beyotim, Shanghai, China). After 15 min, the wells were washed gently with PBS again. Next, 200 μL of 95% ethanol was added to each well to dissolve crystal violet for 10 min, and biofilm thickness was estimated by measuring the OD_595_. The experiment comprised eight within-run replicates.

### 2.6 Animal infection experiments

All experiments were performed according to protocols approved by the Animal Ethics Committee of the Shanghai Veterinary Research Institute, Chinese Academy of Agricultural Sciences (no. SYXK < HU > 2020-0027). In this study, we assessed the relative virulence of WT, WT (*ibaG*), Δ*ibaG* and CΔ*ibaG*. Female ICR mice aged 4 weeks old were obtained from Slack Shanghai Laboratory Animal Co., Ltd., (Shanghai, China), divided into five groups, with six mice in each group, and infected intraperitoneally with *V. parahaemolyticus* (5.0 × 10^7^ CFU per mouse) or PBS as a negative control. After *V. parahaemolyticus* was treated for 48 h, the survival rate of mice was recorded at 1-h intervals. The results were averaged and calculated using the method described by Reed and Muench (1938).

### 2.7 Quantitative bacteriology and histopathology

To further investigate whether *ibaG* gene is associated with severe systemic infection caused by *V. parahaemolyticus*, we used a mouse model of intraperitoneal infection (Matsuda et al., 2019; Lian et al., 2021). No mortality resulted as a consequence of inoculation with 10^7^ CFU. Female ICR mice 4 weeks of age (6 in each group) were challenged using freshly prepared inoculums, WT, WT (*ibaG*), Δ*ibaG* and CΔ*ibaG*, at a concentration of 1 × 10^7^ CFU/mouse for 15 h. The heart, liver, spleen and kidneys of the animals were collected aseptically and placed in 1.5 mL test tubes. All samples were weighed and processed into tissue homogenates using a mechanical homogenizer. A 10-fold dilution of tissue homogenate (100 μL) was plated on TCBS plates to calculate the number of *V. parahaemolyticus* in each sample. Bacteriology data are presented as the number of CFU/g tissue. Heart, liver, spleen and kidney tissue samples (3 in each group) in 4% formaldehyde were submitted to Wuhan Servicebio Technology Co. Ltd. for paraffin processing, embedding and sectioning, following established protocols.

### 2.8 RNA-sequencing

*Vibrio parahaemolyticus* WT and Δ*ibaG* strains were cultured overnight on 3% NaCl-LB agar plates in a three-line method. Single colonies were inoculated into fresh LB medium containing 3% sodium chloride and grown to an OD_600_ of 0.2. Total bacterial RNA was extracted from 1 mL of each strain and treated with TRIzol (Yu et al., 2021). RNA samples were processed using a 4 × gDNA wiper mix (Takara, Shiga, Japan) to remove genomic DNA contamination. RNA sequencing (RNA-seq) was performed on an Illumina HiSeq 2000 platform (Sangon, Shanghai, China), using three replicates of each strain. Differential expression analysis was conducted using the DESeq 2 Bioconductor package.

### 2.9 Quantitative real-time PCR (RT-qPCR)

RT-qPCR was used to further confirm the transcription levels of different expressed genes obtained in RNA-Seq analysis. *V. parahaemolyticus* strains were cultured to logarithmic phase at 37°C. RNA was extracted from 1 mL of each strain according to TRIzol method (Invitrogen, Carlsbad, CA, USA) (Yu et al., 2019). Concentrations of the extracted RNA samples were determined using a NanoDrop spectrophotometer (Thermo Fisher Scientific, Waltham, MA, USA). An equal amount of RNA (1 μg) was reverse transcribed into cDNA according to the concentration measured. RT-qPCR was performed using the Biosystems 7500 Rapid Real-Time PCR System (Foster City, CA). Using 2 μL of cDNA samples diluted 10 times as template, 2X ChamQ Universal SYBR green qPCR main mixture (Takara, Dalian, China) was 10 μL, nuclease-free water 6 μL, and primers 1 μL each. Using the gap gene messenger RNA (mRNA) levels as the criterion, the mRNA levels of each sample were normalized via the 2^–ΔΔCt^. The primers for RT-qPCR are provided in Supplementary Table 1.

### 2.10 Statistical analyses

The GraphPad software package (GraphPad software) was used for statistical analysis and the mapping of data. RNA-sequencing results were analyzed using the DESeq 2 Bioconductor software package to determine the levels of differentially expressed genes.

## 3 Results

### 3.1 Inactivation and growth profile of the *ibaG* gene in *V. parahaemolyticus*

We deleted *ibaG* of 255-bp in the WT strain through homologous recombination. PCR, DNA sequencing analysis (data not shown) and RT-qPCR indicated that the deletion of *ibaG* was successful. The RT-qPCR results showed that the expression of *ibaG* mRNA could not be detected in the deletion strain Δ*ibaG*, while the expression of *ibaG* mRNA could be detected in the complementary strain CΔ*ibaG* constructed by us (Supplementary Figure 1). To assess whether the gene deletion affected the growth of these strains, the growth status of each strain was monitored for 12 h. The results showed that all strains had similar growth spectra under 3% NaCl-LB culture (Supplementary Figure 2) (*P* > 0.05), indicating that the absence of *ibaG* had little effect on *V. parahaemolyticus* growth.

### 3.2 IbaG affects the motility of *V. parahaemolyticus*

**FIGURE 1:**
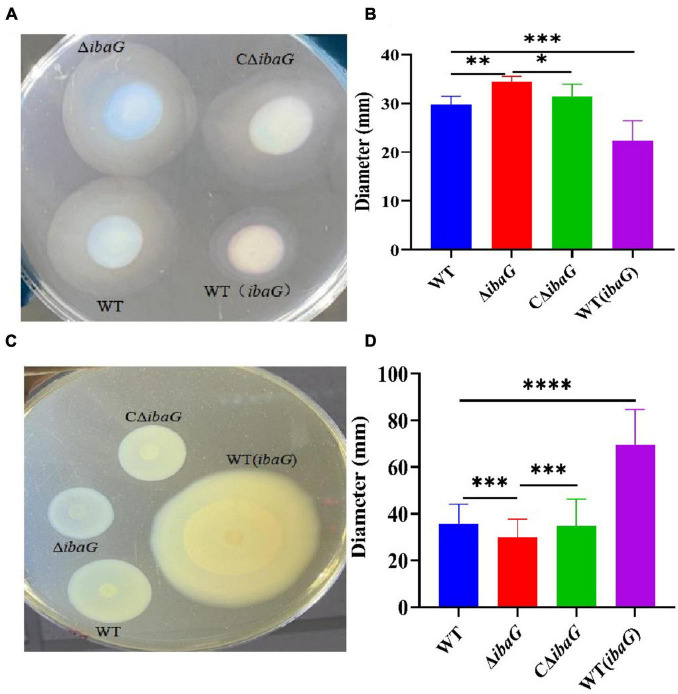
The *ibaG* promotes swarming motility and inhibits swimming motility of *V. parahaemolyticus.* **(A)** To measure swimming motility, bacteria were inoculated in 0.3% agar-2% NaCl-LB plates at 37°C for 4 h. **(B)** Diameter measurements of the swimming motion of each strain. **(C)** The swarming ability of *V. parahaemolyticus* strains determined on 1.5% agar-2% NaCl-HIB medium at 30°C for 12 h. **(D)** Diameter measurements of the swarming motion of each strain.(*P < 0.05, **P < 0.01,***P < 0.001, ****P < 0.0001).

After 4 h of growth in swimming plates, WT, WT (*ibaG*), Δ*ibaG* and CΔ*ibaG* strains all exhibited swimming motility (Figure 1A). The Δ*ibaG* showed the highest swimming motility compared to the other strains WT, WT (*ibaG*) and CΔ*ibaG*. WT and CΔ*ibaG* strains were able to swim more efficiently than WT (*ibaG*) (Figure 1B). The strain Δ*ibaG* produced a 1.10-fold increase in swimming motility relative to WT, whereas WT (*ibaG*) produced a 0.75-fold increase (Figure 1C).

The swarming motility of WT (*ibaG*) was significantly superior to that of the other strains. The colony diameters of WT and Δ*ibaG* were 35 ± 0.75 mm and 30 ± 0.01 mm, respectively. The aggregated area of WT (*ibaG*) was 1.94 times larger than that of WT, whereas the colony diameter of CΔ*ibaG* was similar to that of WT (Figure 1D). In conclusion, *ibaG* has a bearing on the swimming and swarming motilities of *V. parahaemolyticus.*

### 3.3 IbaG contributes to *V. parahaemolyticus* biofilm formation

**FIGURE 2:**
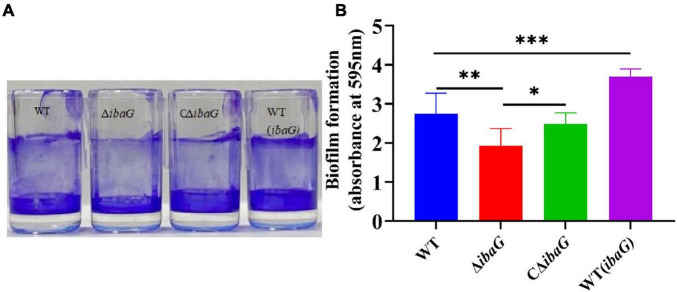
The *ibaG* is required for biofilm formation of *V. parahaemolyticus.* **(A)** The surface-attached cells were stained with crystal violet. **(B)** Crystal violet-stained material was then solubilized in alcohol, and the absorbance at 595 nm was determined. Error bars represent standard deviations. Experiments were conducted in triplica. (*P < 0.05, **P < 0.01, ***P < 0.001).

Biofilm formation was assessed using crystal violet staining to detect the effect of *ibaG* on the biofilm-forming ability of *V. parahaemolyticus* (Figure 2A). Deletion of *ibaG* resulted in a significant reduction in biofilm formation (*P* < 0.05), whereas the biofilm formation of the complementary strain CΔ*ibaG* was restored (Figure 2B), and the biofilm formation ability of the overexpression strain WT (*ibaG*) was significantly enhanced (*P* < 0.05). These results suggest that *ibaG* positively regulates *V. parahaemolyticus* biofilm formation.

### 3.4 Loss of *ibaG* gene attenuated *V. parahaemolyticus* virulence in mice

**FIGURE 3:**
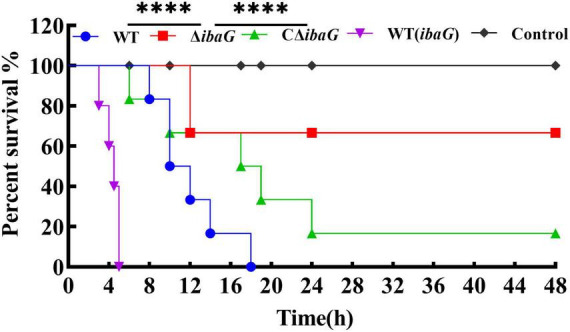
IbaG is responsible for lethality in mice of *V. parahaemolyticus*. The mice infected with 5.0 × 10^7^ CFU of *V. parahaemolyticus* strains were measured to determine the lethality. Mice injected with PBS were used as negative controls. (****P < 0.0001).

To evaluate the comparative virulence of *V. parahaemolyticus* to mice, specific-pathogen-free ICR mice were intraperitoneally inoculated with 5.0 × 10^7^ CFU of *V. parahaemolyticus* strains WT, WT (ibaG), ΔibaG and CΔibaG. The murine survival rate in WT (ibaG) group remained at 0.0%, 5 h after infection (Figure 3). Surprisingly, when ΔibaG was utilized, the lethality in mice (16.7%) was significantly decreased compared with that of the parent strain WT (100%) and the complement strain CΔibaG (83.3%) (Figure 3). These results indicated that ibaG induces lethality in mice.

### 3.5 IbaG helps *V. parahaemolyticus* establish infection in mice

**FIGURE 4:**
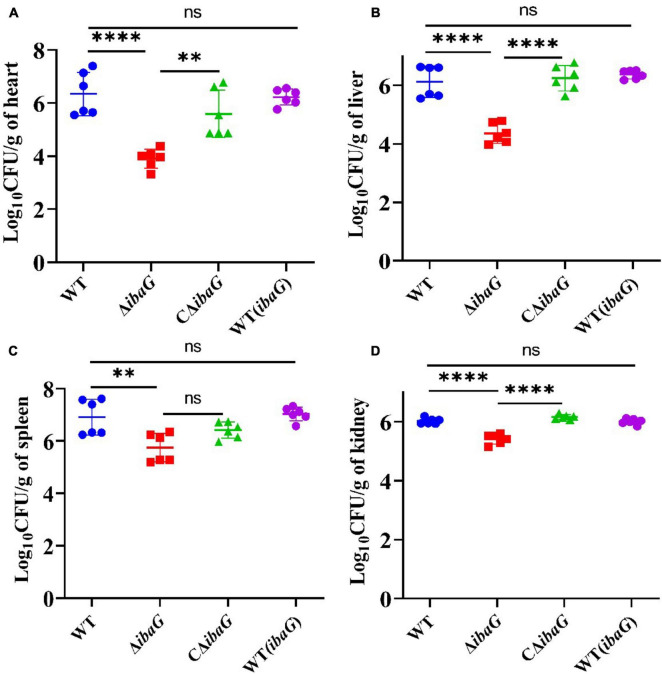
*IbaG* mediates infection in mice induced by *V. parahaemolyticus* Germfree ICR mice were infected with 10^7^ CFU of WT, WT(*ibaG*), Δ*ibaG* and CΔ*ibaG* and euthanized at 15 h p.i. for analysis. Viable bacterial distribution in heart **(A)**, liver **(B)**, spleen **(C)** and kidney **(D)**. (**P < 0.01, ****P < 0.0001).

Intraperitoneally infected 4-week-old ICR mice were administered a bacterial dose of 1 × 10^7^ CFU/mouse for 15 h. Bacterial burden (CFU) in the heart, liver, spleen, or kidney tissues was comparable among *V. parahaemolyticus* strains [WT, WT (*ibaG*), Δ*ibaG* and CΔ*ibaG*]. The *ibaG* mutant strain showed a notable reduction in *V. parahaemolyticus*-induced infections in mice. The bacterial tissue loads in the heart, liver, spleen and kidney of Δ*ibaG* group were 8.87 × 10^3^ CFU/g, 2.9 × 10^4^ CFU/g, 9.77 × 10^5^ CFU/g, 2.73 × 10^5^ CFU/g, respectively. Compared to Δ*ibaG* group, the bacterial tissue loads in WT group were 833-fold higher in heart, 32 -fold higher in liver, 18-fold higher in spleen and 4 -fold higher in kidney, respectively. while no significant differences were found between the bacterial loads from the hearts, livers, spleens and kidneys of the WT, CΔ*ibaG* and WT (*ibaG*) groups at 15 h post bacterial infection (Figure 4). These results suggested that *ibaG* may play an important role in the pathogenesis of *V. parahaemolyticus.*

### 3.6 Loss of *ibaG* gene reduced visceral tissue damage in mice

**FIGURE 5:**
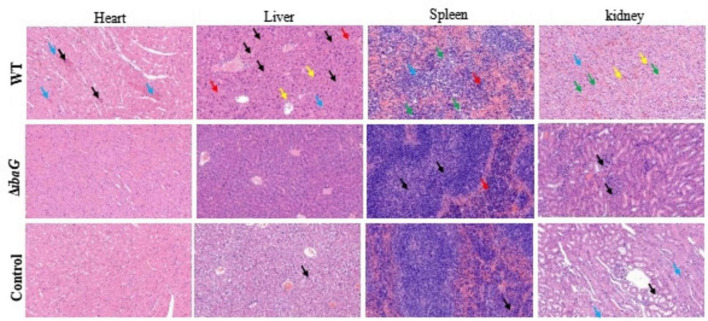
The *ibaG* is conducive to the damage of *V. parahaemolyticus* to the visceral tissues of mice. The images were magnified 200 times. Representative images are presented. Scale bars, 100 μm.

ICR mice were intraperitoneally injected with the *V. parahaemolyticus* strain at a concentration of 10^7^ CFU per mouse and euthanized 15 h after inoculation. The control group was injected with 100 μL PBS. The results of pathological tissue sections of three mice included in each group were basically consistent. Histological examination of tissue sections revealed significant damage to the heart, liver, spleen and kidneys of mice infected with the WT strain. Hemorrhage in the area around the cardiac chamber, marked necrosis of the cardiomyocytes (blue arrows), and scattered blue-purple colonies in the cardiac interstitium (black arrows) were microscopically observed in some cases. The histopathological structure of the liver in the WT group was impaired, hepatic sinusoids (blue arrows) appeared congested, and necrotic hepatocytes were observed (black arrows) (Figure 5). We also observed lymphocytic infiltrates (yellow arrows) and blue-purple colonies (red arrows) in the hepatic sinusoids. These findings indicated that the red pulp of the spleen, including the extramedullary foci of hematopoiesis (red arrows) and necrotic lymphocytes or hematopoietic cells (green arrows), was the area most severely affected by infection. Histopathological evaluation of the kidneys revealed numerous necrotic tubular epithelial cells in the medulla (green arrows), sporadic lymphocytic infiltration into the interstitium (yellow arrows), capillary congestion, and interstitial hemorrhage (blue arrows). Organ lesions observed in mice injected with the WT strain were not observed in mice injected with the *ibaG* mutant strain. Indeed, *ibaG* mutant mice showed markedly reduced histological changes in the heart, liver, spleen and kidney compared to mice injected with the WT strain.

### 3.7 RNA-Seq analysis identifies DEGs in *ibaG* mutants

**FIGURE 6:**
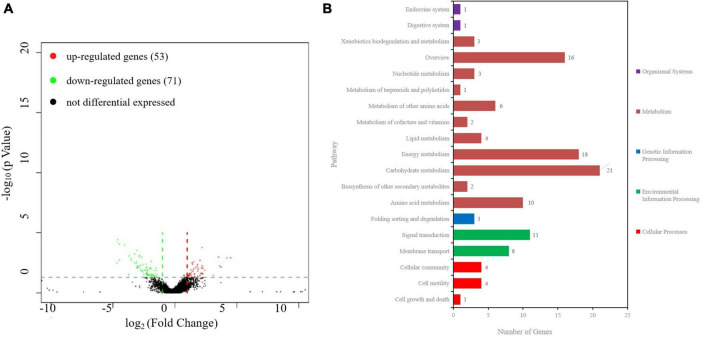
RNA sequencing analysis of *ibaG* and and WT strains of *V. parahaemolyticus*. **(A)** A volcano plot was generated to visualize the differentially expressed genes in Δ*ibaG*. The x-axis represents the log2 of the fold change gene expression between different groups of samples. Red and green points indicate the up- and downregulated genes, respectively. **(B)** KEGG analysis of the pathways in Δ*ibaG*. The number on each bar represents the number of differentially expressed genes in each pathway.

**FIGURE 7:**
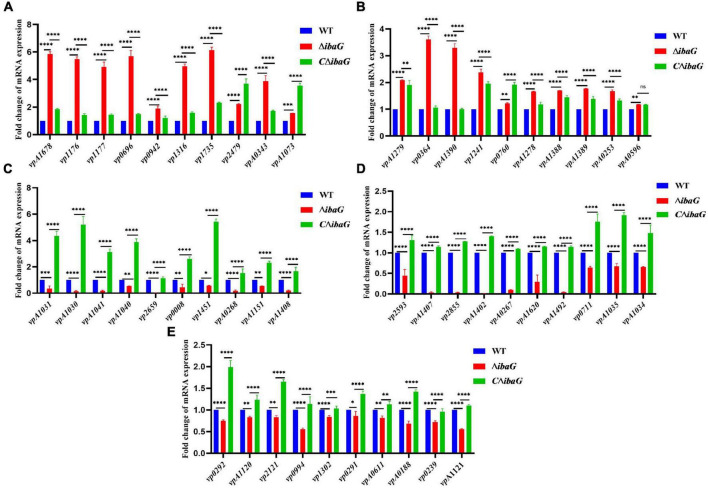
**(A–E)** Real-time PCR verification of differentially expressed genes in strains WT, Δ*ibaG* and *C*Δ*ibaG*. Data were normalized to the housekeeping gene *gap*. Results are shown as relative expression ratios compared to expression in the WT strain. Stars represent significant differences (*P < 0.05, **P < 0.01, ***P < 0.001, ****P < 0.0001).

To examine the genes affected by ibaG, we performed RNA-seq with total RNA extracted from WT and ΔibaG in triplicate for each strain to obtain a detailed transcription profile. A comparison between the transcriptions of *V. parahaemolyticus* WT strain and ΔibaG mutant (log2FC > 1 or log2FC < −1, corrected *P*-value < 0.05) revealed a total of 4,534 overlapping co-expressed genes. IbaG regulated the expression of 124 DEGs, of which 71 were downregulated and 53 were upregulated (Figure 6A). To further explore the function of these DEGs, KEGG pathway analysis was performed, indicating that six genes related to the type VI secretion system were downregulated, and several genes related to energy and amino acid metabolism were affected (Figure 6B). Finally, to determine the accuracy of the RNA-seq results, we examined the expression levels of 50 randomly selected DEGs via RT-qPCR. The results showed that the expression levels of 50 DEGs were consistent with those of the RNA-seq analysis, indicating the reliability of the RNA-seq results (Figures 7A–E). The mRNA expression values of some genes in complemented strain CΔibaG did not restore to the wild type levels might due to that CΔibaG was constructed by the introduction of the ibaG gene into the ΔibaG mutant using a complementation vector, and thus differential gene dosages as well as plasmid maintenance during gene transcription.

## 4 Discussion

In recent years, the number of *V. parahaemolyticus* infections has increased in many countries. This is due to numerous virulence factors linked to *V. parahaemolyticus*, which seriously endanger the global aquaculture industry as well as public safety and health (Nair et al., 2007). BolA, which is considered to be closely associated with the virulence of pathogens, such as S. Typhimurium and *Klebsiella pneumoniae*, promotes bacterial survival under harsh conditions (Mil-Homens et al., 2018). In *E. coli, bolA* is a well-established stress-response gene, but the role of its homologue in *V. parahaemolyticus* is yet to be assessed. In this study, we performed the first systematic analysis of *vp2659* (255-bp encoding protein IbaG) in the genome of *V. parahaemolyticus* SH112. We successfully constructed the Δ*ibaG* (255-bp) mutant and obtained its revertant *C*Δ*ibaG* and overexpression WT (*ibaG*) to confirm its function in the pathogenesis of *V. parahaemolyticus*.

*V. parahaemolyticus* has a double flagellar system suitable for movement in diverse situations. Polar flagella promote swimming motility in liquid environments, and lateral flagella drive the movement of bacterial populations on semi-solid surfaces, facilitating resistance to adverse environmental conditions and access to nutrients (Grognot et al., 2023). Motility contributes to the adhesion and invasion of bacteria into host cells during the pathogenic process, which is conducive to bacterial pathogenicity (Yang et al., 2020). A previous study showed an inhibitory effect on bacterial swimming in a semi-solid medium, owing to impaired flagellar assembly in BolA-overexpressing strains (Vieira et al., 2004; Dressaire et al., 2015). Therefore, we can speculate that the swimming ability of bacteria increases in the absence of *bolA*, whereas motility is significantly reduced in strains overexpressing *bolA* (Moreira et al., 2017). In the present study, we observed decreased swimming motility of the *ibaG* overexpressing strain WT (*ibaG*) and increased motility by mutation of the *ibaG* gene in *V. parahaemolyticus*, when compared to the WT. Furthermore, we found that *ibaG* deletion resulted in a defect in swarming, whereas overexpression of this gene stimulated swarming of *V. parahaemolyticus.* These results reveal that IbaG in *V. parahaemolyticus* SH112 might regulate motility-related cellular processes in a manner separate from other BolA-like regulators of *V. parahaemolyticus*.

Biofilm formation is an evolutionary adaptation seen in microbial communities that enables bacteria to survive and spread in austere environments (Zhong et al., 2021). Biofilms prevent antibiotics from infiltrating bacterial cells, thereby increasing bacterial resistance, environmental adaptability, and tolerance (Rather et al., 2021; Wang et al., 2023). Biofilm formation, which is a well-studied but extremely complex process, requires the participation and cooperation of multiple cellular structures and components and involves five distinct stages (Stoodley et al., 2002). Previous studies have shown that the transcription factor BolA, which facilitates the switch between planktonic and fixative lifestyles, plays a key role in biofilm formation in a variety of organisms (Silva et al., 2020). BolA is involved in biofilm formation not only in *E. coli* (Vieira et al., 2004; Adnan et al., 2010) but also in *Pseudomonas fluorescens* (Koch and Nybroe, 2006). In the case of *V. parahaemolyticus*, our results indicated that biofilm formation by the Δ*ibaG* strain was weaker than that by the WT strain and *ibaG* overexpression led to an increase in biofilm production. Similar studies that focused on other pathogenic bacteria reported that the capacity for biofilm formation was significantly reduced due to the lack of *bolA*, whereas overexpression of *bolA* significantly increased biofilm formation, thereby substantiating the validity of our results (Vieira et al., 2004). Exopolysaccharides play a key role in biofilm maturation, and the *VPA1403-1412* (*cpsA-J*) operon is responsible for exopolysaccharide production in *V. parahaemolyticus* (Zhang et al., 2018). The RNA-seq results of this study showed that *cpsA-F* transcription levels were down-regulated in *ibaG* mutants compared to that in the WT. We hypothesized that IbaG may affect *V. parahaemolyticus* biofilm formation by reducing the number of exopolysaccharides. This study demonstrated that the expression of a BolA-like protein, IbaG, contributes to increased biofilm formation.

Pathogenic bacteria have evolved various mechanisms to coordinate genes that regulate different synergistic and antagonistic effects to better survive in the environment as well as in human hosts (Hou et al., 2019; Pazhani et al., 2021). *V. parahaemolyticus* can cause gastroenteritis, in severe cases, once the bacterium enters the host, it can further spread into the blood, resulting in systemic sepsis and lesions of internal organs (Baker-Austin et al., 2018; Lian et al., 2021). When cultures were used to infect mice, the *ibaG* mutant exhibited a sizable deficit in *V. parahaemolyticus* virulence relative to that of the WT strain. The mutant also displayed impaired capacity to colonize the heart, liver, spleen and kidney in an animal infection model. Compared to the WT, *ibaG* deficiency alleviated the damage to mouse internal organs (heart, liver, spleen and kidney), as characterized by significant necrosis of myocardial cells, congested hepatic sinusoids, lymphocyte infiltration, extramedullary hematopoietic foci, and much necrotic renal tubular epithelial cells in the medulla. More specifically, the Δ*ibaG* strain reduced infection of mice compared with the WT strain. Mil-Homens et al. (2018) demonstrated that BolA plays an important role in *S. Typhimurium* pathogenesis (Vieira et al., 2004). For the first time, the BolA-like protein, IbaG, was found to be associated with virulence and colonizing capacity of *V. parahaemolyticus.* Multiple virulence factors regulate the pathogenicity of *V. parahaemolyticus*. At present, several studies, exploring the basic mechanisms underlying *V. parahaemolyticus*-related infections, are underway. However, studies aimed at elucidating the genetic mechanisms underlying the reaction between this pathogen and host cell infection remain scant. Therefore, more detailed molecular mechanistic studies are required to explain the pathogenesis of *V. parahaemolyticus* (Pazhani et al., 2021).

RNA-seq results showed that 53 genes were upregulated and 71 genes were downregulated in the mutant strain Δ*ibaG*, which altered various pathways, including those related to bacterial secretion system, two-component system, ABC transporter system, quorum sensing and amino acid metabolism. Studies conducted on *V. cholerae* have shown that IbaG interacts directly or indirectly with several proteins involved in iron-sulfur biogenesis or iron-sulfur clusters. Iron-sulfur containing proteins play key roles in cellular processes, such as central carbon metabolism and signal transduction, suggesting that *ibaG* deficiency may disrupt cellular physiological processes in multiple ways (Azam et al., 2020). In our study, *ibaG* gene deletion decreased the expression of *vp1451*, encoding a 4Fe-4S binding protein. It is speculated that *ibaG* may affect the cell physiological process of bacteria by influencing the expression of *vp1451*, but the specific mechanism needs to be further explored. The type VI secretion system (T6SS) is a macromolecular complex that facilitates the delivery of effector proteins to eukaryotic and bacterial cells. It comprises an inner tube made of stacked Hcp hexamer rings, which is engulfed in a sheath that includes two subunits, TssB and TssC (Douzi et al., 2018). Six T6SS proteins, TssA, TssE, TssF, TssG, TssK and VgrG, are required for the proper assembly of Hcp tubes (Brunet et al., 2015). In *Acinetobacter baumannii*, TssL is a cytoplasmic protein that binds to the inner membrane to form membrane complexes that anchor the transport system to the bacterial cell wall (Ruiz et al., 2020). The expression of *tssB*, *tssC*, *tssE*, *tssF*, *tssK* and *tssL* was downregulated following *ibaG* deletion, suggesting that IbaG may regulate bacterial secretion by affecting the essential component proteins of T6SS. In addition, *vpA1151* and *vp2479*, which are associated with ABC transporters, were found to be downregulated and upregulated, respectively. ABC transporters are associated with the outer membrane biogenesis of gram-negative bacteria (Narita, 2011). *VP0008* expression was downregulated, similar to *Bacillus subtilis* YxeM, which plays a role in L-cystine uptake. VmeG and VmeH belong to the RND (Resistance, Nodulation and cell Division) efflux transporter family, the members of which may function in nodulation, acriflavin resistance, heavy metal efflux, or multidrug resistance. Loss of *ibaG* resulted in the upregulation of *vmeG* and *vmeH*. These lines of evidence suggest that members of the BolA family act not only as transcriptional regulators but also form a complex system that regulates multiple metabolic processes and modulates the expression of numerous genes.

In the present study, we found that IbaG in *V. parahaemolyticus* SH112 is involved in multiple biological processes, including swarming motility, swimming motility, biofilm formation, bacterial colonization and mouse virulence, demonstrating for the first time that the transcriptional regulator IbaG (*vp2659*) acts as an important virulence factor. Our data suggested that IbaG may affect the virulence of *V. parahaemolyticus* by participating in numerous cellular metabolic processes, such as motility and biofilm formation. Understanding the role of IbaG may help manage the contamination and clinical infections caused by *V. parahaemolyticus*. However, the mechanism through which IbaG affects various phenotypes has not been fully elucidated. Thus, a wide range of experiments aimed at investigating the mechanisms regulating *ibaG* are warranted.

## Data availability statement

The datasets presented in this study can be found in online repositories. The names of the repository/repositories and accession number(s) can be found in the article/Supplementary material.

## Ethics statement

The animal study was approved by the Animal Ethics Committee of the Shanghai Veterinary Research Institute, Chinese Academy of Agricultural Sciences approved all animal infection studies (no. SYXK 2020-0027). The study was conducted in accordance with the local legislation and institutional requirements. The studies were conducted in accordance with the local legislation and institutional requirements. Written informed consent was obtained from the owners for the participation of their animals in this study.

## Author contributions

WW: Formal analysis, Investigation, Writing–original draft. YL: Formal analysis, Investigation, Writing–original draft. SL: Validation, Writing–original draft. PL: Validation, Writing–original draft. XH: Methodology, Writing–review and editing. WS: Formal analysis, Writing–review and editing. QW: Methodology, Writing–review and editing. WF: Investigation, Writing–review and editing. WJ: Conceptualization, Formal analysis, Funding acquisition, Supervision, Writing–review and editing.
