# Supplementary material for: BolA-like protein (IbaG) promotes biofilm formation and pathogenicity of Vibrio parahaemolyticus
Source: Front Microbiol. 2024 Jul 31;15:1436770. doi: 10.3389/fmicb.2024.1436770 (PMC11322356; doi:10.3389/fmicb.2024.1436770)
Supplement: Supplementary file 1 [file Table_1.DOCX]

Supplementary Table 1

List of primers used in RT-qPCR.

| **Number** | **Name** | **Sequence** |
| --- | --- | --- |
| 1 | *vp1241*-RT-F | TGCTTACGGCAATGATGTTGACG |
| 2 | *vp1241*-RT-R | TGCGCGGCTCTTGGATTAGTTTC |
| 3 | *vpA1390*-RT-F | GCCGAGCGAGGGAGTTAAAACG |
| 4 | *vpA139*0-RT-R | TTGCCACTGCTGCTATCTTCTTCC |
| 5 | *vp0364*-RT-F | GCGGTGTTGGTGGATGACGATG |
| 6 | *vp0364*-RT-R | ACGAGCCAGTGCTTCACATTGC |
| 7 | *vpA1279*-RT-F | TCAGCGATGACTGGCATGAACTC |
| 8 | *vpA1279*-RT-R | CTAAAGCAAGAACGACGCCCAAAC |
| 9 | *vpA1073*-RT-F | TGCCTACACAACCGCCTACCG |
| 10 | *vpA1073*-RT-R | GTGAGCCATGCCAACCAGTATCG |
| 11 | *vpA0596*-RT-F | TGTTGGCGAGTTGGGTGAGATTTC |
| 12 | *vpA0596*-RT-R | CTGCCCTTGCCGCTTCTATCG |
| 13 | *vpA0343*-RT-F | GAAGTCTGCTCAAGCCGCTCTC |
| 14 | *vpA0343*-RT-R | ACAAACTGACCCACACCGACATC |
| 15 | *vp2479*-RT-F | CTACGGTGGCGGTACAGTGAATG |
| 16 | *vp2479*-RT-R | TGTTGTGAGTTGCTTCGTCAGACC |
| 17 | *vp1735*-RT-F | GCTCTCGGTTGGCTTGTTAGTCAG |
| 18 | *vp1735*-RT-R | GGACAGCGTCAGCAGCATCAG |
| 19 | *vp1316*-RT-F | GAGGCTGGCACTCAGTCTATTCG |
| 20 | *vp1316*-RT-R | GCTGCTGAACAAATGGTCCGTTTC |
| 21 | *vp0942*-RT-F | GCGGGTATTATGGCGTTGTTAGTC |
| 22 | *vp0942*-RT-R | GCGGAAAACTTTTCTGCGGTCTC |
| 23 | *vp0696*-RT-F | GAGATGGCTATGCGGTTGGGTAC |
| 24 | *vp0696*-RT-R | GGAAGGCGTTACTGCGTTTGTTG |
| 25 | *vp1177*-RT-F | GCGTGATGGTGCGAGAAGGG |
| 26 | *vp1177*-RT-R | CGAGAGCGGCGATATTGACTGTC |
| 27 | *vp1176*-RT-F | GTGGTTGCGAAAGCCGACATTAC |
| 28 | *vp1176*-RT-R | ACCCAAGCCCGAACTTTGATTCC |
| 29 | *vpA1678*-RT-F | TGATGTGGTGAGCAAGCCGTTAG |
| 30 | *vpA1678*-RT-R | TGAGGCGGACAGGCAGGTG |
| *31* | *vpA1408*-RT-F | *TTACAACCGACGCAGAGATTACCC* |
| *32* | *vpA1408*-RT-R | *CCGAGGCATGAACTTCACCAATTC* |
| *33* | *vpA1407*-RT-F | *GTCTGCTCAAGCGAAGCGTTATG* |
| *34* | *vpA1407*-RT-R | *ACCAATCGGCTCTCTCACCAATG* |
| *35* | *vp2593*-RT-F | *GGCGTGGAAGTAGCGGGTAAAC* |
| *36* | *vp2593*-RT-R | *CGACCGTGTTCAGGCAGAGATAC* |
| *37* | *vp2855*-RT-F | *ATCAGCAACATCCAAGACGGCATC* |
| *38* | *vp2855*-RT-R | GCACGAGTTTCACGACCTGTAGC |
| 39 | *vpA1402*-RT-F | ACACGATCAGGTGGAAGCAATGAC |
| 40 | *vpA1402*-RT-R | GCGGTTCTGTGGGTAGTGGTAAAG |
| 41 | *vpA0267*-RT-F | GCCACAACATTGCCAACGCTTC |
| 42 | *vpA0267*-RT-R | CTTCTACGCCGCCAGGTTGC |
| 43 | *vpA1620*-RT-F | AGTGCTTTACCCGAACGACAACC |
| 44 | *vpA1620*-RT-R | GTACAGACGCTGCTGAGTGGAAG |
| 45 | *vpA1492*-RT-F | GCAGTAATGACGCAAGCAGAAGC |
| 46 | *vpA1492*-RT-R | AGCAGAGGTCGCATTGTCACTTAC |
| 47 | *vpA1151*-RT-F | AGCAGGTCGCCCAGAGTCTTG |
| 48 | *vpA1151*-RT-R | CGCCGTTGACGATCCCGATAAC |
| 49 | *vpA0268*-RT-F | GTCCGTTGGTGGTGCCTGATTAC |
| 50 | *vpA0268*-RT-R | CAATGTGCCGACTGCGATTTCTG |
| 51 | *vp1451*-RT-F | GCGGTGAGTGTAAGTCGGCATG |
| 52 | *vp1451*-RT-R | GCATCGTATGGGCAACTGTCCTC |
| 53 | *vp0008*-RT-F | AGCCGCAACTGAAGTCAAGGTG |
| 54 | *vp0008*-RT-R | CGTTACGCTTACCGATCTCATCCC |
| *55* | *vpA1041*-RT-F | AAGCGGACACAACAACGGATGAG |
| 56 | *vpA1041*-RT-R | GGCACGACACTTAAAGGAGCTACG |
| 57 | *vpA1040*-RT-F | TGGCAACGGAGATTGGCAAGG |
| 58 | *vpA1040*-RT-R | ACAACGCTCGCACTACCAGATTC |
| 59 | *vpA1030*-RT-F | GCGGGCTACCAAAGGTTCAGTTAC |
| 60 | *vpA1030*-RT-R | TGCCATCGTGTGCTTTCATCCATC |
| 61 | *vpA1031*-RT-F | TCGGGAGTTTGAACCTCGGTTAAG |
| 62 | *vpA1031*-RT-R | GGCTCGACTTCCGAATTGAATCTC |
| *63* | *vpA1034*-RT-F | AGAGGGTGGTGGGCGAGTTG |
| 64 | *vpA1034*-RT-R | GCTTCACGGCGGTCTGTAATGC |
| 65 | *vpA1035*-RT-F | CGGAAGCCTCGTGTTCACATTACC |
| 66 | *vpA1035*-RT-R | AGTCGCCCATAACACCCACCAC |
| 67 | *vp0711*-RT-F | AAGGCACGGGCGACATTAAAGG |
| 68 | *vp0711*-RT-R | GGAGACTGATACACTGGCGTTAGC |
| 69 | *vpA0253*-RT-F | CCTCATTGTGACCTTCTTGACTACG |
| 70 | *vpA0253*-RT-R | GTTCATTGCCATTGCTGCTTCTG |
| 71 | *vpA1389*-RT-F | TGTTGCGTTGGGTATGAAAGAGG |
| 72 | *vpA1389*-RT-R | TCGGCTCCGTAAGGTACACAG |
| 73 | *vpA1388*-RT-F | GTACCAACATCACCACTTAGACTCC |
| 74 | *vpA1388*-RT-R | ACCCATACGGAAGACTCTGTGTAG |
| 75 | *vpA1278*-RT-F | GCCAAGGTTACGCAGAGAAAGTC |
| 76 | *vpA1278*-RT-R | GCACCGCCAATACCATCTTCAAG |
| 77 | *vp0760*-RT-F | TCTCCAATGTACGCAGACAAGTTC |
| 78 | *vp0760*-RT-R | CAGTAATCATCACCAGCACCTAGAC |
| 79 | *vp0292*-RT-F | GTGCGATTGAATCTGAAGCGAATAC |
| 80 | *vp0292*-RT-R | ACCTTGACGTTTCTTGAGTTCCATC |
| 81 | *vp0239*-RT-F | AAGTAATCATCCAATACGGCGGTTC |
| 82 | *vp0239*-RT-R | CGATGTCTGGTTGTGAGAAGTAAGC |
| 83 | *vp1302*-RT-F | AAGCGGCTCAATCTATCGTTATGC |
| 84 | *vp1302*-RT-R | CCAAGTAGGTTTAGGATGTCGGATG |
| 85 | *vpA0188*-RT-F | GAATACGCCAGCAGGTGTTCC |
| 86 | *vpA0188*-RT-R | ATGTCGTACTTGGTCCACAACTTG |
| 87 | *vpA0611*-RT-F | GCGGTATTGGCGAGAACTCTATG |
| 88 | *vpA0611*-RT-R | GCACCTAGCAACTCAGATGTAGC |
| 89 | *vp0291*-RT-F | TGGCAACGAACGATTCAGTCAC |
| 90 | *vp0291*-RT-R | AATGCTACTTCGCCTGGTTTGAG |
| 91 | *vp0994*-RT-F | AAGGTAATGGAAGGTATCAAGCAAGAG |
| 92 | *vp0994*-RT-R | TCGTGAGCAGTGATGGTAGAGATG |
| 93 | *vp2121*-RT-F | ACTTCCGTCGTGGTAGCCTTC |
| 94 | *vp2121*-RT-R | TCTGCGTAACCGTTGTTGAATAGG |
| 95 | *vpA1120*-RT-F | CATTCGCATCGCAGAAGAACAAG |
| 96 | *vpA1120*-RT-R | GTCACACGCTCACCACACAAG |
| 97 | *vp2659*-RT-F | TGGTTGCTGTTGATGCTTGTTTCG |
| 98 | *vp2659*-RT-R | CTTATCACGAGCCCACTCTTCCG |
| 99 | *vpA1121*-RT-F | GTTGGCTATTCGGTTGCAGT |
| 100 | *vpA1121*-RT-R | CGATGGCGGCAATAAACTAT |
