# Supplementary figures and images for: BolA-like protein (IbaG) promotes biofilm formation and pathogenicity of Vibrio parahaemolyticus
Source: Front Microbiol. 2024 Jul 31;15:1436770. doi: 10.3389/fmicb.2024.1436770 (PMC11322356; doi:10.3389/fmicb.2024.1436770)

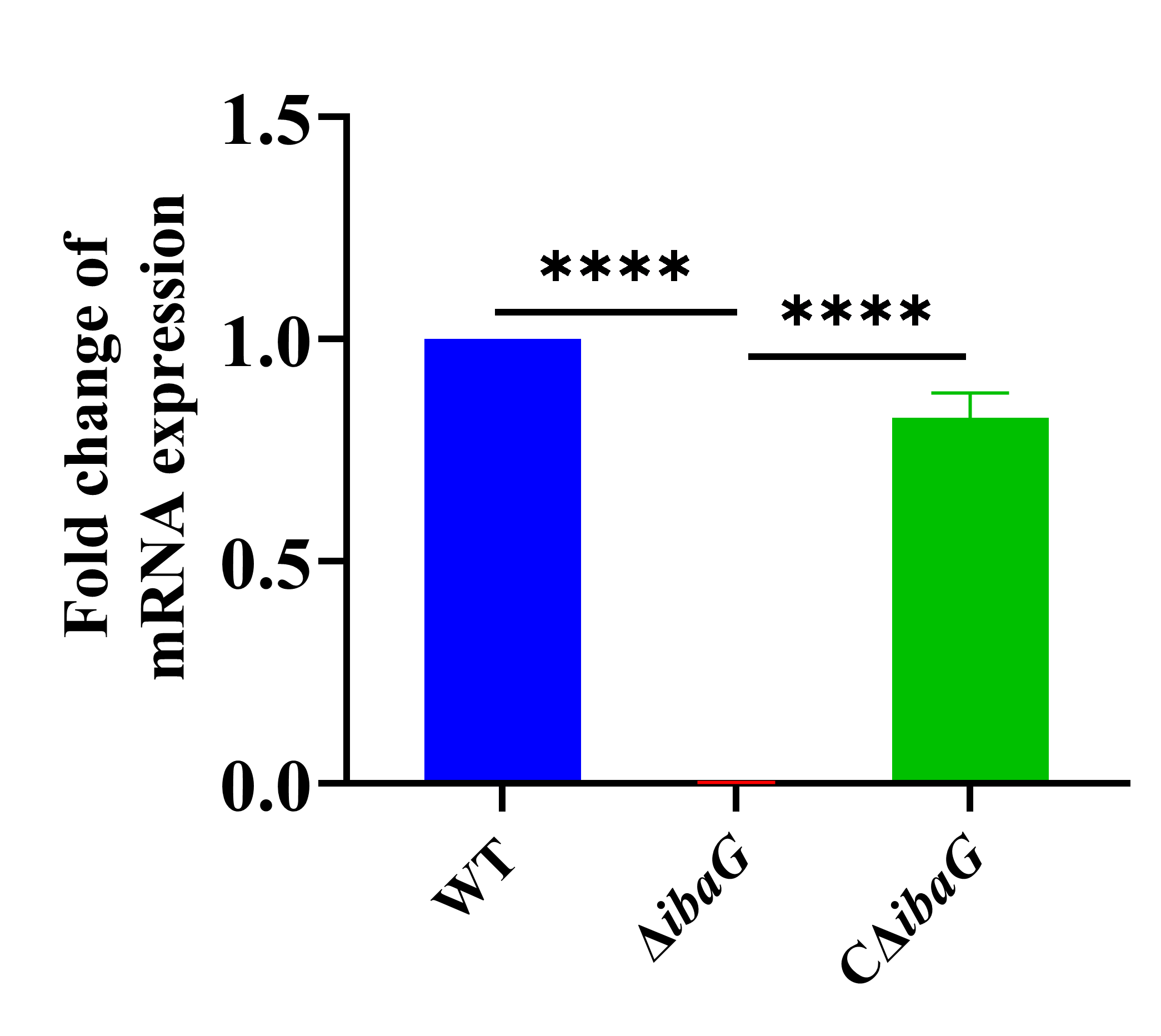

Supplement: Supplementary file 2 [file Image_1.TIF]

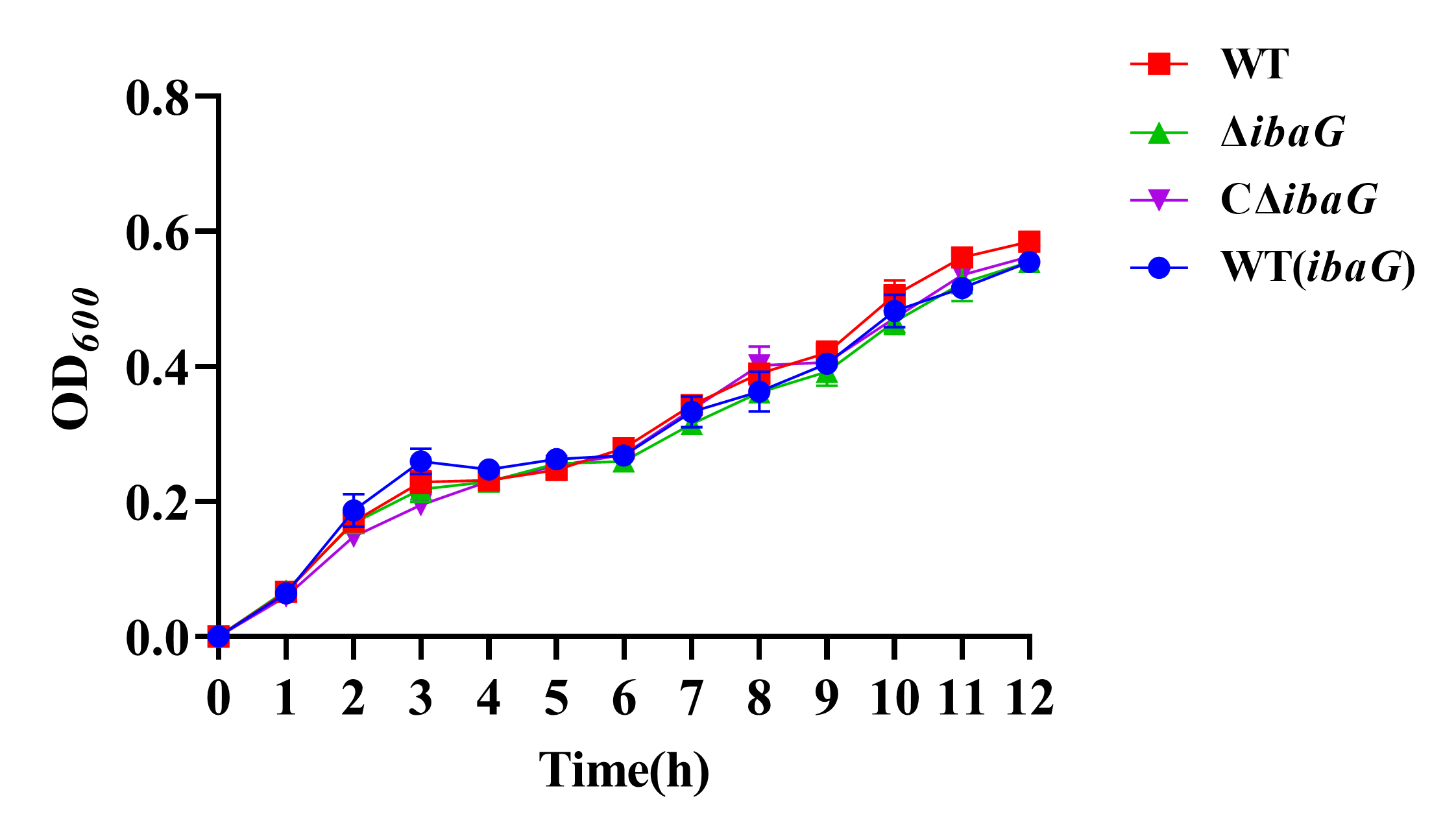

Supplement: Supplementary file 3 [file Image_2.TIF]
